# Assessing REM Sleep as a Biomarker for Depression Using Consumer Wearables

**DOI:** 10.3390/diagnostics15192498

**Published:** 2025-10-01

**Authors:** Roland Stretea, Zaki Milhem, Vadim Fîntînari, Cătălina Angela Crișan, Alexandru Stan, Dumitru Petreuș, Ioana Valentina Micluția

**Affiliations:** 1 Department of Neurosciences, Psychiatry and Pediatric Psychiatry, Faculty of Medicine, Iuliu Hațieganu University of Medicine and Pharmacy, 400012 Cluj-Napoca, Romania; roland.miha.stretea@elearn.umfcluj.ro (R.S.); milhem.zaki@umfcluj.ro (Z.M.); ccrisan@umfcluj.ro (C.A.C.); imiclutia@umfcluj.ro (I.V.M.); 2 Wakez Health Tech Ecosystem SRL, 400686 Cluj-Napoca, Romania; vadim@steepsoft.com (V.F.); petreusdumitru@gmail.com (D.P.); 3 Clinical Emergency Hospital for Children, 400370 Cluj-Napoca, Romania

**Keywords:** depression, REM sleep, apple watch, machine learning, digital biomarker

## Abstract

**Background:** Rapid-eye-movement (REM) sleep disinhibition—shorter REM latency and a larger nightly REM fraction—is a well-described laboratory correlate of major depression. Whether the same pattern can be captured efficiently with consumer wearables in everyday settings remains unclear. We therefore quantified REM latency and proportion of REM sleep out of total sleep duration (labeled “REM sleep coefficient”) from Apple Watch recordings and examined their association with depressive symptoms. **Methods:** 191 adults wore an Apple Watch for 15 consecutive nights while a custom iOS app streamed raw accelerometry and heart-rate data. Sleep stages were scored with a neural-network model previously validated against polysomnography. REM latency and REM sleep coefficient were averaged per participant. Depressive severity was assessed twice with the Beck Depression Inventory and averaged. Descriptive statistics, normality tests, Spearman correlations, and ordinary-least-squares regressions were performed. **Results:** Mean ± SD values were BDI 13.52 ± 6.79, REM sleep coefficient 24.05 ± 6.52, and REM latency 103.63 ± 15.44 min. REM latency correlated negatively with BDI (Spearman ρ = −0.673, *p* < 0.001), whereas REM sleep coefficient correlated positively (ρ = 0.678, *p* < 0.001). Combined in a bivariate model, the two REM metrics explained 62% of variance in depressive severity. **Conclusions:** Wearable-derived REM latency and REM proportion jointly capture a large share of depressive-symptom variability, indicating their potential utility as accessible digital biomarkers. Larger longitudinal and interventional studies are needed to determine whether modifying REM architecture can alter the course of depression.

## 1. Introduction

Sleep is a reversible physiological state characterized by specific neurophysiological patterns, alternating throughout the night between two stages: non-rapid eye movement (NREM) and rapid eye movement (REM) sleep [1]. Research shows that REM sleep is involved in memory consolidation, emotional processing, and neuroplasticity [2,3]. Interactions between several brain circuits enable NREM-REM cycling throughout sleep. Monoaminergic neurons are least active during REM sleep, while cholinergic neurons increase their activity during REM sleep [4,5,6]. Disturbances in these circuits are consistently linked to psychiatric conditions such as depression [7].

Depression, also known as major depressive disorder (MDD), is a serious psychiatric illness marked by low mood, anhedonia and cognitive and somatic symptoms [8]. Its pathophysiology, among other mechanisms, involves monoaminergic dysregulation, with abnormalities highlighted in regions also involved in sleep–wake control [9,10]. It is a leading cause of disability worldwide, affecting an estimated 280 million people worldwide, with a rise in prevalence by nearly 28% since 1990 [11].

Current approaches, albeit efficient, often fail to account for individual variability. Diagnosis remains clinical, with no objective evaluation available and little to no use of biomarkers other than laboratory work to rule out organic or other medical causes [12]. Nevertheless, several candidate biomarkers show promise in research. Elevated cortisol levels have been linked to mechanisms involved in depressive pathophysiology [13], blood levels of pro-inflammatory markers are consistently higher in depression and partially normalize with effective treatment [14], and peripheral BDNF is typically reduced in acute depressive episodes and rises with response and remission [15].

Sleep architecture also shows promise as a candidate biomarker: certain parameters, such as total duration spent in REM sleep and REM latency, have been associated with depression across literature [16,17,18]. Current advances in technology support a shift towards “digital phenotyping”: consumer wearables collect sensor data regularly, at scale and reliably, and evidence suggests that clinical models based on collected data have potential to improve detection of at-risk individuals [19].

This study was designed to evaluate whether two wearable-derived features of sleep architecture, namely REM latency (REM-L) and the proportion of REM sleep (labeled as “REM coefficient”, REM-C), are associated with depressive symptomatology. Our objective was to quantify these associations in everyday conditions using multi-night smartwatch recordings and symptom scores, and to explore their potential utility for risk stratification and early identification, without advancing mechanistic claims.

## 2. Materials and Methods

### 2.1. Participants

A total of 191 adults took part in the study, comprising 80 men and 111 women, all older than 18 years. Age distribution was as follows: 49 participants were 18–25 years old, 66 were 26–30 years old, 55 were 31–40 years old, 13 were 41–50 years old, and 8 were 51–60 years old. All respondents were permanent residents of Romania at the time of enrolment; the great majority (153) lived in urban areas, while 38 resided in rural communities. Educational attainment was diverse: 66 held a bachelor’s degree, 82 had completed postgraduate studies, and 43 were high-school graduates. Regarding marital status, 115 participants were unmarried, 40 were living in cohabitation, and 36 were married. Professional status varied: 118 were full-time employees, 27 identified as freelancers, 18 were entrepreneurs, 22 were students, and 6 were unemployed at the time of data collection.

### 2.2. Procedure

A team of researchers from “Iuliu Hațieganu” University of Medicine and Pharmacy conducted a prospective, observational, cross-sectional association study, between January and June 2025. The study was conducted in accordance with the Declaration of Helsinki, and approved by the Institutional Review Board of the Department of Public Health, “Babeș-Bolyai” University, Cluj-Napoca (protocol code IRB#2024-241218-001, date of approval 23 December 2024). Assessment of depressive symptoms, together with detailed sleep-architecture metrics, was conducted using technological means, in a digital environment, through a proprietary application running on Apple (Apple Inc., Cupertino, CA, USA) software. The equivalent of a TRL 5 integrated digital system consisting of an iOS mobile application and a WatchOS application, in TestFlight (extended beta) regime, were provided by the company involved in the development of the application, free of charge for research purposes, as well as for participants in the study.

Recruitment relied on a single Google Forms questionnaire disseminated via email lists and social-media channels. Eligible volunteers were adults who understood English and either owned an iPhone and/or Apple Watch or had at least minimal experience and ability in using smartphone environments. In order to widen access and alleviate data skewing, the team provided several Apple Watch units to participants who might otherwise be unable to afford hardware.

Individuals were excluded if they declined or were unable to give informed consent, carried comorbid sleep disorders such as chronic insomnia or narcolepsy, or reported other significant chronic medical conditions (e.g., cardiovascular or pulmonary disease). Furthermore, shift-workers were excluded because of their unpredictable sleeping schedules and certain medication users were refused participation because of the effects on sleep architecture of said medicine. Informed consent was obtained from all subjects involved in this study.

A custom algorithm and mobile platform were utilized for sleep staging. The system uses a supervised machine learning pipeline to process smartwatch sensor data, with model training and execution managed on AWS SageMaker.

While multiple supervised learning models were evaluated, including logistic regression, k-nearest neighbors, and random forest, a multilayer perceptron (MLP) neural network consistently achieved the best performance and was selected for the final system. The classification pipeline follows a two-stage approach:

- A binary MLP (Multi-Layer Perceptron) classifier first detects sleep versus wake epochs (wake/sleep).
- Epochs classified as sleep are then processed by a multi-class classifier (also MLP) to identify REM versus NREM sleep stages (REM/NREM).

The models are trained using raw sensor data collected from an Apple Watch, specifically the triaxial MEMS accelerometer and photoplethysmography (PPG) heart rate sensor, supplemented by auxiliary time-based data and modeled circadian trends. To ensure clinical accuracy, ground-truth labels were obtained from concurrent, professionally scored polysomnography (PSG), gold standard for sleep measurement. All sensor data and PSG labels are segmented into 30-s, non-overlapping epochs to align with standard sleep scoring protocols.

To ensure robustness and prevent overfitting, the models are trained and validated using a 5-fold stratified cross-validation scheme. The primary validation metric is the Area Under the Receiver Operating Characteristic Curve (AUC), which effectively measures the model’s ability to distinguish between classes. Performance is also assessed using precision, recall, and F1 scores. Further information on the reliability, validity and general accuracy of our AI algorithm and model is presented in the Discussions section. More extensive documentation regarding our approach is provided in the Supplementary Materials (Document S1).

Participants enrolled in the study were instructed to wear an Apple Watch for 15 consecutive nights while using the proprietary mobile platform. Sleep-related data were collected via both the Apple Health framework and the custom platform. Multiple parameters were calculated, including the total duration of sleep, total REM sleep duration, the number of REM episodes during the whole night, and notably, the REM latency (REM-L)—defined as the elapsed time from sleep onset to the first detected REM period. To assess depressive symptomatology, the Beck Depression Inventory II (BDI-II, from here on referenced as BDI) was administered twice via the same mobile application: once at baseline (Day 0) and again at the end of the tracking period. The BDI, a 21-item survey validated for evaluating depression severity, yields scores ranging from 0 to 63, with higher values indicating greater symptom severity [20]. For analysis, the mean of the two BDI scores was used to represent each participant’s depressive symptom level.

Following data acquisition, all variables were compiled into a single anonymized dataset, identified solely by unique user identifiers in accordance with GDPR compliance. A fully anonymized table containing all sleep-related data and survey scores, with corresponding user identifiers, assigned automatically through the application for each participant, appears in the Supplementary Materials.

The primary variables of interest for statistical analysis were the average BDI score (referred to through the “BDI” variable), the average REM sleep duration expressed as a percentage of total nightly sleep—REM coefficient, referred to as the “REM_C” variable, and the average REM-L of the user, referred to as the “REM_L” variable.

Following data curation, descriptive statistics, including means, medians and standard deviations, were performed for all primary variables. Outlier detection was done using the 1.5 × interquartile range rule, and distributional assumptions were assessed with Kolmogorov–Smirnov and Shapiro–Wilk tests, associated with visual inspection of histograms and Q-Q plots. Associations between primary variables were analyzed through Spearman’s rank correlation coefficient. Linear regression analyses were conducted via the Ordinary Least Squares method (OLS), with model assumptions evaluated through Q-Q plots of residuals and Breusch-Pagan tests for homoscedasticity. Robust HC3 standard errors were applied where heteroscedasticity was detected, in order to ensure stable inference. Exploratory covariate analysis (by sex) was conducted. All tests were two-tailed, with a significance threshold set at an alpha value of 0.05. Bonferroni correction was conducted to mitigate multiple-testing bias. All analyses were performed using IBM^®^ SPSS^®^ v26.0.0.

Generative-AI tools were employed exclusively for post-production tasks—namely spelling, grammar and syntax correction as well as graphic layout and generation of illustrative images. No generative system was used to create or modify the scientific text, interpretations or statistical analyses presented in this manuscript.

## 3. Results

### 3.1. Descriptive Statistics

**Figure 1:**
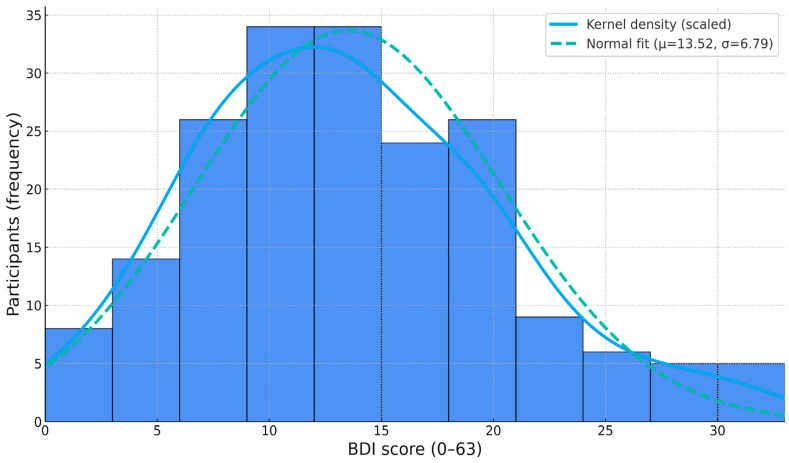
Histogram of BDI score averages.

**Figure 2:**
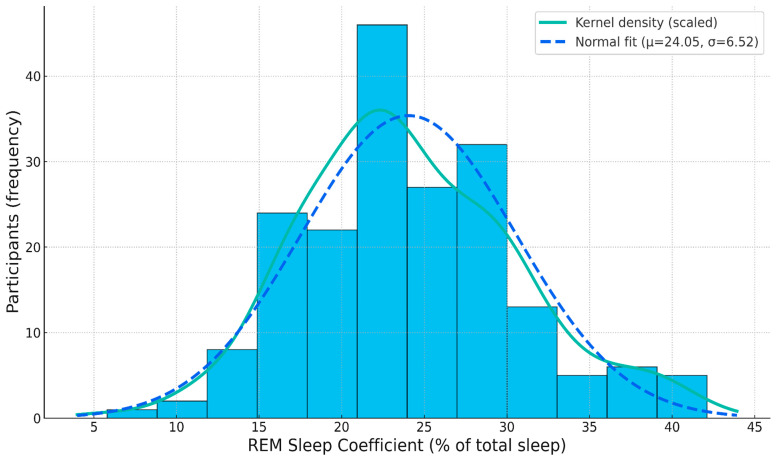
Histogram of REM_C.

**Figure 3:**
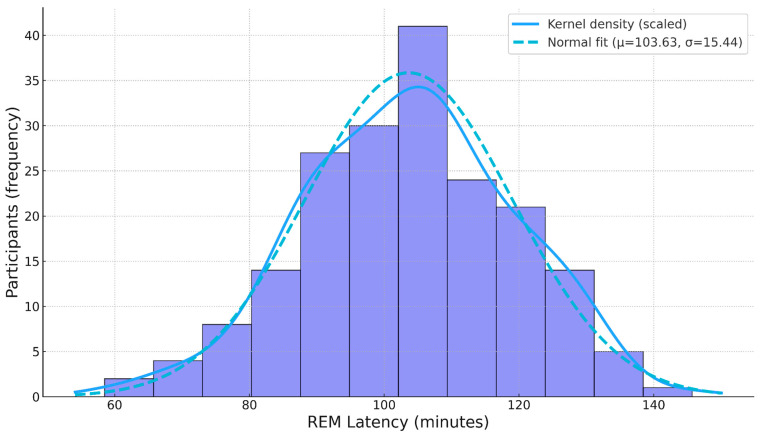
Histogram of REM_L.

Across the cohort, the mean BDI score was 13.52 (SD = 6.79, median = 13), with values ranging from 0 to 33. Participants exhibited an average REM_C of 24.05 (SD = 6.52, median = 23.29), spanning values from 5.80 to 42.11. REM_L averaged 103.63 min (SD = 15.44 min, median = 104.1 min), with observations between 58.5 min and 145.7 min. Histograms for each variable are shown in Figure 1, Figure 2 and Figure 3.

### 3.2. Correlation Analysis

#### 3.2.1. Outlier and Normality Tests

We began by screening the dataset for extreme observations using the conventional 1.5 × interquartile-range rule. Neither BDI scores nor the two sleep variables displayed values beyond the upper or lower fences, so all cases were retained. Distributional assumptions were examined with Kolmogorov–Smirnov and Shapiro–Wilk tests. Kolmogorov–Smirnov testing showed no departure from normality for any of the three measures. Shapiro–Wilk test confirmed normality for REM_L and was marginal for REM_C, but indicated a modest deviation for BDI. Consequently, we made use of rank-based testing for correlations in order to reduce errors to a minimum.

#### 3.2.2. BDI—REM_L Correlation (Figure 4)

Given scientific evidence that major depression is accompanied by a smaller REM_L, we tested the hypothesis that REM_L would correlate inversely with BDI. Using the raw minute values, Spearman’s rho was −0.673, *p* < 0.001, showing that participants who had lower REM_L reported higher BDI scores. To demonstrate that the coding direction does not drive the result, we generated a reflected variable (ΔREM_L = mean(REM_L) − REM_L) so that higher numbers represent shorter latencies; the correlation became +0.673 with an identical *p*-value.

**Figure 4:**
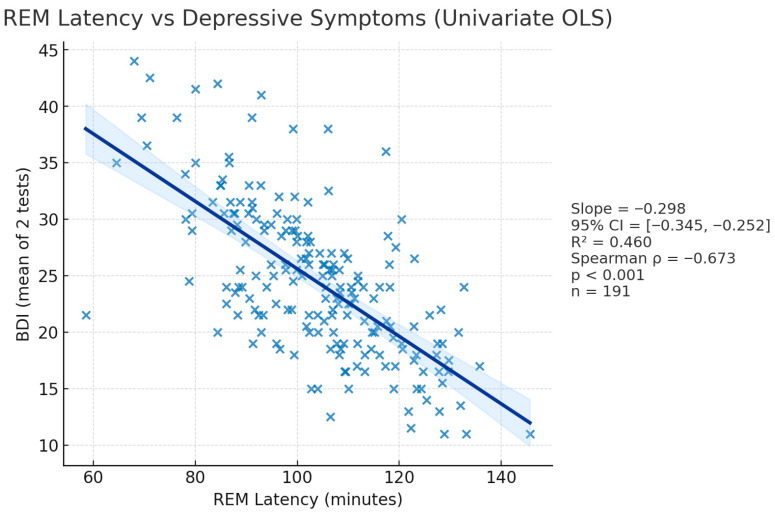
Scatterplot displaying REM_L—BDI association.

#### 3.2.3. BDI—REM_C Correlation (Figure 5)

Existing evidence tells us that patients with depressive disorders typically spend more time during REM sleep, and as such we tested the hypothesis that REM_C correlates directly with BDI. Following analysis, REM_C showed a robust positive association with BDI − rho = 0.678, *p* < 0.001.

**Figure 5:**
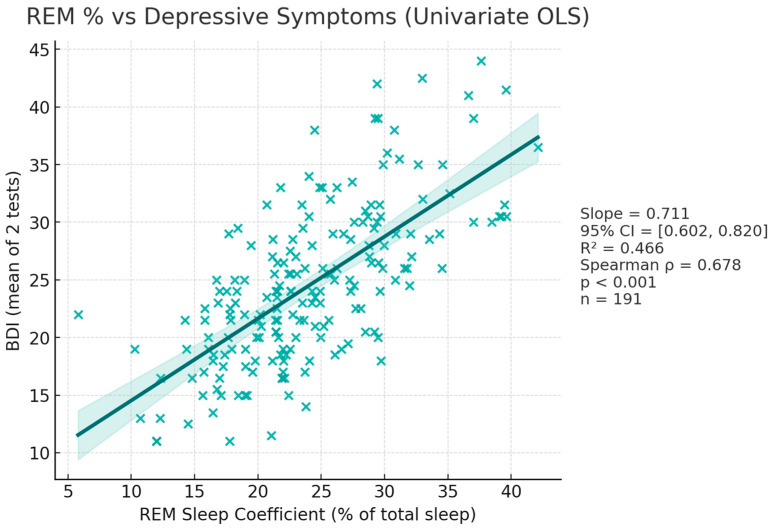
Scatterplot displaying REM_C—BDI association.

### 3.3. Linear Regression

A series of Ordinary Least Squares regressions were estimated to quantify how REM sleep metrics account for variability in depressive severity. All analyses used the raw minute scale for REM-L (so that a negative slope represents the theoretical inverse relationship) and the averaged BDI score as the outcome.

As key findings, in the univariate models, REM-L alone accounted for 46% of the variance in BDI. Every 1 min reduction in REM-L predicted a 0.3 point rise in BDI scores (β = –0.298, 95% CI = –0.35 to –0.24, *p* < 0.001) (Figure 4). REM_C accounted for 46% of the variance. Each 1 percent increase in REM_C predicted a 0.71 increase in BDI scores (β = 0.711, 95% CI = 0.6 to 0.83, *p* < 0.001) (Figure 5). Entering both predictors simultaneously increased the explained variance to 62% and left both slopes highly significant (REM_L: β = –0.200, 95% CI = −0.244 to −0.156, *p* < 0.001; REM_C: β = 0.482, 95% CI = 0.376 to 0.587, *p* < 0.001), indicating independent contributions.

**Figure 6:**
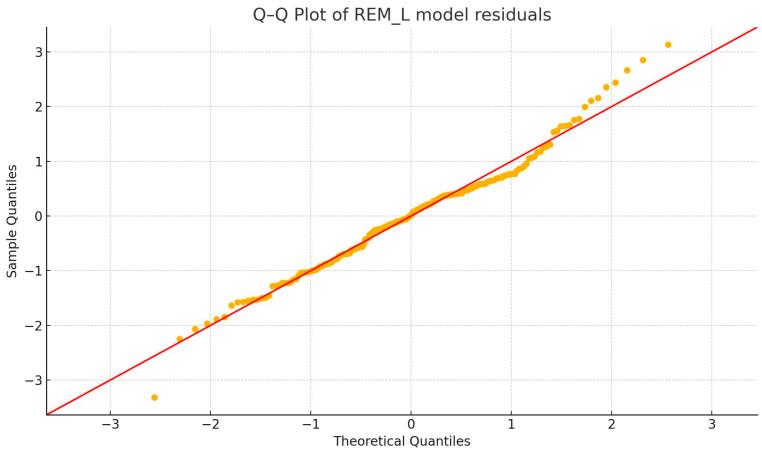
Q-Q plot of REM_L model residuals.

**Figure 7:**
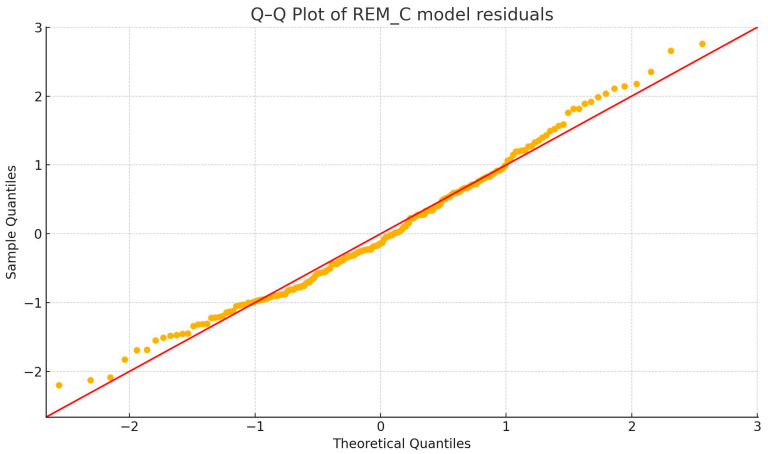
Q-Q plot of REM_C model residuals.

**Figure 8:**
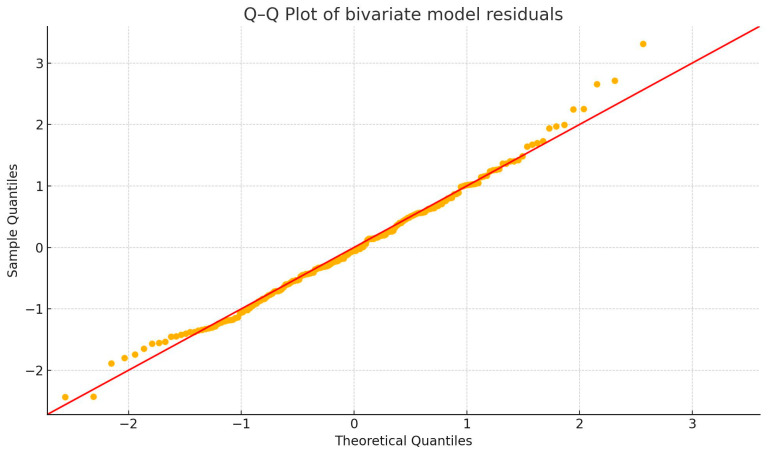
Q-Q plot of bivariate model residuals.

Visual inspection of Q–Q plots showed the residuals to be approximately normally distributed in all three models. Homoscedasticity was evaluated with the Breusch–Pagan test; *p*-values were 0.008 for the REM_L model, 0.139 for the REM_C model, and 0.105 for the bivariate model. Thus, modest heteroscedasticity was detected when REM_L acted alone, but the issue attenuated when REM_C was included. Robust standard errors (HC3) were therefore computed as a sensitivity analysis and left all *p*-values and confidence intervals materially unchanged, confirming the stability of the inferences. Q-Q plots are available below (Figure 6, Figure 7 and Figure 8).

### 3.4. Covariate Analysis

To partly mitigate confounders, sex was included as a binary covariate (0 = male, 1 = female) in an exploratory analysis. Rank-based associations between sleep markers and depressive symptoms were strong overall and within each sex. For REM_L, stratified values were ρ = −0.623 in males (*p* < 0.001) and ρ = −0.731 in females (*p* < 0.001). For REM_C, values were ρ = 0.697 in males (*p* < 0.001) and ρ = 0.664 in females (*p* < 0.001). Ordinary least-squares models adjusting for sex confirmed that these relationships were not explained by sex differences. In BDI ~ REM_L + sex, the slope for REM_L was −0.296 BDI points per minute (*p* < 0.001) while the sex term (female vs. male) was not significant (β = −0.804, *p* = 0.276); R^2^ = 0.464. In BDI ~ REM_C + sex, the slope for REM_C was +0.706 BDI points per 1 point REM_C (*p* < 0.001), with a non-significant sex term (β = −0.559, *p* = 0.448); R^2^ = 0.467. When both sleep variables were entered simultaneously (BDI ~ REM_L + REM_C + sex), each retained an independent association with BDI (REM_L β = −0.200 per minute, *p* < 0.001; REM_C β = +0.479 per 1 point REM_C, *p* < 0.001), and sex again remained non-significant (β = −0.351, *p* = 0.572); model R^2^ = 0.625. Interaction terms did not indicate materially different slopes by sex (REM_L × sex β = +0.090, *p* = 0.057; REM_C × sex β = −0.084, *p* = 0.456).

### 3.5. Multiple Testing Correction

Two primary hypotheses were prespecified (Spearman ρ between BDI and REM_L; between BDI and REM_C). Additional association tests (univariate and multivariable OLS slopes for REM_L and REM_C) were considered secondary/sensitivity analyses. To most conservatively control the family-wise error rate, Bonferroni correction was applied across the six reported tests, after which all associations remained statistically significant (all adjusted *p* < 0.001). To address multiplicity with sex in the model (sex coded 0 = male, 1 = female), we applied Bonferroni correction consistently across related test sets. The correction was used for: sex-adjusted main effects of REM latency and REM proportion on BDI, considering both the single-predictor models and the joint model; sex-by-effect interaction terms in the joint model; and sex-stratified Spearman correlations (males and females, each for REM latency–BDI and REM proportion–BDI). After analysis and adjustment, all main-effect *p*-values and all sex-stratified correlation *p*-values remained statistically significant (*p* < 0.001). The interaction terms did not meet the Bonferroni-adjusted significance threshold, indicating no evidence of sex-specific effect modification.

## 4. Discussion

This study examined REM sleep architecture in relation to depressive symptoms among adults without formal diagnoses. Shorter REM-L and greater REM-C were each strongly associated with higher BDI scores; together they explained over 60% of between-person variance. Clinically speaking, in our study, a 10-min reduction in REM-L corresponded to an expected 3-point increase in BDI, whereas a 5% rise in REM-C corresponded to a 3.6-point increase. When both REM characteristics were considered together, they explained nearly two-thirds of individual differences in symptom scores.

Our findings mirror the well-established REM signature of depression reported by earlier polysomnographic studies [16,17] and confirmed by research groups across years of research and different cohorts [21,22,23]. Furthermore, this stand-alone study extends our earlier investigation, where REM-C alone showed a strong correlation with BDI scores (Spearman ρ ≈ 0.70; *p* < 0.001), explaining nearly half of the variance [18]. The notion that REM sleep could serve as an objective biomarker for depression has deep historical roots. REM abnormalities observed repeatedly in depression fit with models displaying cholinergic predominance alongside monoaminergic down-regulation during REM sleep, together with heightened limbic sensitivity and altered prefrontal control. Converging neuroimaging and systems-level evidence shows that REM selectively augments amygdala–hippocampal responsivity and modulates medial prefrontal control during affective processing, a configuration thought to support overnight emotional recalibration [24]. Exploratory functional data further link REM physiology to mood regulation. Human fMRI shows that a night rich in REM sleep is followed by reduced amygdala reactivity to prior emotional stimuli (“overnight affective depotentiation”), whereas disrupted REM leaves hyperreactivity intact. In depression, excess/early REM may consolidate negative salience and blunt extinction-like processes, offering a mechanistic route from REM disinhibition to higher symptom burden [25]. Pharmacologic data support this potential bidirectional link, without establishing definitive causality: antidepressant pharmacotherapies frequently used in MDD (e.g., SSRIs, SNRIs, activating TCAs) increase REM-L and decrease REM-C, whereas more sedating agents (e.g., mirtazapine, trazodone) may improve continuity and slow-wave sleep while still modifying REM regulation [26], demonstrating that monoaminergic tone can interfere with REM architecture.

As to other directions and sides to this link, experimental manipulations of REM sleep, including selective REM deprivation, have been explored extensively in order to highlight their potential in the management of affective disorders. In one of the earliest studies in the field, selective REM deprivation produced rapid symptomatic improvement [27], while Cartwright’s longitudinal studies found out that spontaneous REM reductions predicted better patient outcomes [28]. More recently, REM-suppressing pharmacologic strategies have shown antidepressant potential [29]. Experimental manipulations, including selective sleep/REM deprivation, can transiently improve mood in a subset of patients [30,31].

These neurobiological and experimental accounts provide context for interpreting associations, but mechanistic or causal claims are beyond the scope of this observational study. Our current contribution aims to demonstrate the feasibility and scalability of estimating REM-L and REM-C from consumer wearables in naturalistic, multi-night conditions and to quantify their independent associations with depressive symptom severity. These findings extend prior PSG-based literature by showing that similar association patterns can be recovered at home and at scale, in ecological settings, towards future prospective and more clinically anchored validation.

A notable feature of our cohort was the high rate of clinically relevant symptoms despite no formal diagnoses: nearly 1 in 5 displayed clinically relevant depressive symptoms, with 32 individuals (17%) fell in the moderate BDI band (20–28), and 5 (3%) reached the severe band (≥29). This is consistent with community screens—especially in student populations [32]. The sleep characteristics of many participants, otherwise formally undiagnosed, elicited the classical pattern of REM sleep disinhibition, supporting the idea that REM changes may prove useful in early screening or risk monitoring contexts [17].

We also discuss our methodological and practical approaches. We recorded 15 nights of sleep in 191 participants, with little to no data loss, exceeding previous examples of stable home sleep estimates [33]. Our sample size far exceeds known research. At this observed effect size, our design delivers enough power to detect associations at an alpha of 0.05. Regarding validity, data collected by an Apple Watch is considered well-suited for our purpose. Apple Watch sensors are FDA-cleared, and sleep/REM performance sits near the top among consumer wearables. Apple’s own four-stage sleep-classification algorithm achieves a sleep–wake sensitivity of 97.9% and a specificity of 75%, and delivers epoch-level REM detection that approaches 77% accuracy when benchmarked against laboratory polysomnography [34]. A 2024 review places the Apple Watch performance near the top of a wide spectrum of consumer wearables [35], effectively achieving “state-of-the-art” status. Our in-house model, previously validated against laboratory polysomnography, correctly labelled 93% of sleep epochs and distinguished REM from non-REM sleep with an accuracy of 72% [36]. The MESA dataset was and is used as a benchmark for testing and validation [37,38]. Given these performance metrics, the technology is “good enough for purpose” in an epidemiological context: the stage-level accuracy exceeds the 70% threshold that the American Academy of Sleep Medicine cites as acceptable for home-based staging research [39]. Nevertheless, a <80% accuracy is prone to help surface residual misclassifications. People with MDD or more prominent depressive symptoms tend to exhibit higher sleep fragmentation in the form of lower sleep efficiency and higher wake-after-sleep-onset [40], which are more “unpredictable” models of sleep in the eyes of a prediction-based staging model. However, from an epidemiologic standpoint, what we analyzed is actually a “proxy” with various degrees of noise for the true REM parameter values—a “classical” error model. Correlations and regression slopes are expected to be biased toward the null (regression-dilution), meaning our effect sizes are likely conservative underestimates. Multi-night averaging further reduces random error, enhances ecological validity and reduces laboratory “first-night” effects. Finally, our findings are also to be viewed as proof-of-concept for machine-learning-based sleep staging in mental health contexts, rather than definitive diagnostic applications.

Study limitations include selection bias, namely toward younger, urban, higher-educated participants with higher socio-economic status, traits reflected by Apple device ownership. Studies indicate that urban living amplifies physiological and neural responses to psychosocial stressors [41,42], helping to explain both the higher prevalence of mood disorders compared to rural regions and the high apparent “undiagnosed” percentage of urbanites in our sample. Furthermore, socioeconomic status could influence health literacy and study adherence: adults with lower income or lower education levels are more likely to have inadequate health literacy—predicting poorer self-management of chronic conditions and lower self-awareness about health status [43]. Device ownership patterns further constrain recruitment [44]; future studies should intentionally broaden recruitment. To limit potential confounders introduced by demographic data, we included an exploratory covariate analysis. We did not include age, marital status or occupation as exploratory covariates for several reasons. First, our cohort was concentrated in early- to mid-adulthood, where normative age effects on REM architecture are small per decade and pronounced shifts occur in late life [45,46]; within such a restricted age range residual confounding is expected to be minimal and adjustment risks unnecessary loss of precision. Second, marital status and occupation primarily index socioeconomic position and psychosocial context; these factors are upstream determinants of depressive symptoms and general sleep quality, often acting through mood rather than directly altering REM microarchitecture. In longitudinal data, associations between socioeconomic status and sleep quality were fully mediated by depressive symptoms, indicating that conditioning on such variables can block portions of the total association of interest [47].

An important aspect to keep in mind is translation to clinical populations. Many patients with clinical depression have sleep conditions (such as insomnia or sleep apnea), other comorbidities that could alter sleep quality and medical treatments that modify sleep architecture. For example, as shown before, data indicate that primary insomnia is associated with disruptions of sleep continuity and reductions in REM sleep alongside reduced slow-wave sleep, suggesting a baseline architecture that differs from community samples [40]. Prevalent comorbidities in clinical practice (such as cardiometabolic disease, anxiety disorders, substance use and chronic pain) can further perturb REM dynamics and interact with wearable staging performance. Effect sizes and any candidate thresholds derived here should be taken with a grain of salt. Future research should intentionally stratify participants according to traits known to interfere with REM sleep.

A key limitation is the cross-sectional, short-term nature of the design. Because exposure and outcome were assessed within the same window, temporal ordering cannot be established; therefore, the observed associations should not be interpreted as causal. Future work should prioritize longitudinal and interventional designs and test whether REM changes precede symptom worsening; as studies suggest, adults and adolescents who display reduced REM-L after recovery are more likely to relapse [48,49].

One final limitation is reliance on the BDI itself, a validated, but self-reported questionnaire, with no structured diagnostic interviews or clinician-rated assessments; as such, our findings pertain to depressive symptom severity rather than a full-blown disorder. Furthermore, self-report measures can introduce recall and mood-congruent reporting biases. Under classical non-differential error, variability in the BDI-II primarily inflates residual variance and tends to attenuate correlations and narrow apparent effect sizes; Systematic bias correlated with sleep complaints (e.g., negative affectivity) could also distort associations. Future studies should incorporate multi-method case evaluation to strengthen construct validity and support clinical translation.

Through both this present study and the aforementioned previous research we conducted, we claim little advancement to mechanistic hypotheses. Our contribution lies in the recognition of the potential of consumer wearables in mental health—strongly emphasizing its role in allowing individuals to actively track their mental health with agency. Although no single sensor can “diagnose” depression, the ensemble of nightly REM measures, daytime HRV trends, and activity regularity captured by the Apple Watch could form a credible physiological proxy for mood state, especially when aggregated over weeks [50], supporting feasibility and scalability in using consumer wearables as health trackers [51]. The urgency of such proactive monitoring is amplified by the current geopolitical climate: peer-reviewed surveys document sharp spikes in stress and anxiety linked to armed conflicts, economic instability, and climate-related threat, with downstream increases in depressive symptoms across entire populations [52,53].

## 5. Conclusions

The present study shows that two REM sleep parameters captured by Apple Watch—shortened REM latency and increased REM duration—are associated with higher depressive symptom scores. These findings strengthen the case for using objective REM metrics as candidate digital biomarkers for depressive symptomatology, risk stratification and monitoring. This research illustrates that a readily available consumer device could gather sleep data detailed enough to detect potentially clinically relevant patterns, without establishing a definitive causal link. Future interventional studies are needed to determine whether deliberate modification of REM architecture affects symptom outcomes.

## Data Availability

A dataset containing all relevant and curated sleep data and BDI score averages is available in the Supplementary Materials.

## Supplementary Materials

The following supporting information can be downloaded at: https://www.mdpi.com/article/10.3390/diagnostics15192498/s1: Document S1: Machine learning documentation. Table S1: Dataset.xls.
