# Supplementary material for: Assessing REM Sleep as a Biomarker for Depression Using Consumer Wearables
Source: Diagnostics (Basel). 2025 Oct 1;15(19):2498. doi: 10.3390/diagnostics15192498 (PMC12523841; doi:10.3390/diagnostics15192498)
Supplement: Supplementary file 1 [file diagnostics-15-02498-s001.zip › Document S1.pdf]

# Machine learning documentation

Our sleep staging algorithm utilizes a supervised machine learning pipeline to classify sleep stages from raw wearable sensor data. The system is designed to be robust, transparent, and backward-compatible with legacy data formats, drawing upon established methods for feature extraction and validation. The pipeline involves distinct stages for data preparation, feature engineering, and model training, which are detailed below.

While several classifiers including Logistic Regression, k-Nearest Neighbors, and Random Forest were evaluated, a **multilayer perceptron (MLP) neural network**, implemented using the `scikit-learn` library, consistently achieved the best performance for both sleep-wake and multi-class sleep stage classification. Additional architectures, such as LSTM or Transformer architectures, are trained on every new data batch (new sleep sessions recorded), as generalization capabilities might change in time. The (current) final production system employs the MLP architecture.

The classification process follows a two-stage approach: first, a binary classifier detects **sleep vs. wake** epochs. Second, epochs classified as sleep are passed to a multi-class classifier to distinguish between **REM and NREM** sleep stages. The system automatically detects the required feature set (19 features) based on the input layer size of the trained model, ensuring seamless compatibility between legacy and enhanced versions.

The models are trained using raw sensor data collected from consumer wearable devices (Apple Watch), time-aligned with ground-truth labels from concurrent polysomnography (PSG). The raw data consists of triaxial accelerometry (motion) and photoplethysmography (PPG) signals used to derive heart rate. All data is segmented into **30-second non-overlapping epochs** to match standard PSG scoring protocols.

The feature set is supported to maintain both legacy compatibility and enhanced performance. It contains 19 features and provides a richer representation of the underlying physiological state for improved accuracy. It includes:

- **10 Motion Features:** Statistical and temporal features derived from the accelerometer, including total motion, mean, standard deviation, max, min, percentiles, zero-crossings, and range.

- **8 Heart Rate Features:** Advanced metrics derived from the PPG signal, including mean, standard deviation, min, max, percentiles, and metrics inspired by heart rate variability (HRV).
- **1 Circadian Feature:** The same cosine-transformed time value used in the legacy model.

To ensure robustness and prevent overfitting, models are trained and validated using a **5-fold stratified cross-validation** scheme. This method maintains the original distribution of sleep stages in each fold, which is critical for imbalanced datasets.

The primary validation metric is the **Area Under the Receiver Operating Characteristic Curve (AUC)**, which effectively measures the model's ability to distinguish between classes across all possible thresholds. For multi-class (Wake/NREM/REM) classification, a one-vs-rest AUC score is computed.

To be promoted for production use, trained models must pass stringent quality gates based on their cross-validation performance:

- **Sleep/Wake Classifier:** Must achieve a mean AUC of ≥ **0.80**.
- **REM Classifier:** Must achieve a mean AUC of ≥ **0.75**.

This rigorous, automated training and validation pipeline ensures that only high-performing, generalizable models are deployed.
